# Inhibitory event-related potentials during soccer-specific decision making in collegiate players

**DOI:** 10.3389/fspor.2026.1972760

**Published:** 2026-10-19

**Authors:** Takahiro Matsutake, Hiroki Nakata, Yumiko Yamaguchi

**Affiliations:** 1 Research Center for Urban Health and Sports, Osaka Metropolitan University, Osaka, Japan; 2 Faculty of Engineering, Nara Women’s University, Nara, Japan; 3 Faculty of Health Sciences, Kansai University of Health Sciences, Osaka, Japan

**Keywords:** event-related potentials, inhibitory control, lower-limb, pass-canceling, soccer

## Abstract

Inhibitory control is a critical component of executive function that may contribute to athletic performance. However, neurocognitive research in soccer has largely relied on general, nonsport-specific tasks. Therefore, this study examined behavioral and neurophysiological indices of inhibitory control, specifically the N2 and P3 components of event-related potentials (ERPs), using visual, auditory, and soccer-specific Go/No-go tasks, and investigated their relationships with physical fitness and technical–tactical performance in amateur collegiate soccer players. Forty-one male collegiate soccer players participated in the study, with analytic sample sizes varying across assessments because of technical problems, artifact rejection, and physical constraints. Participants performed the Go/No-go tasks using a foot-switch response while electroencephalographic activity was recorded. Physical fitness was assessed using a 30-m sprint test, the Arrowhead Agility Test, and the Yo-Yo Intermittent Recovery Test Level 2 (Yo-Yo IR2). Technical–tactical performance was evaluated during 4-vs-4 small-sided games using the Game Technical Scoring Chart (GTSC). During the soccer-specific task, reaction time was significantly associated with the peak amplitude of the subtracted No-go-N2 at Fz, with shorter reaction times associated with more negative No-go-N2 amplitudes (*r* = 0.552, *p* < 0.001, *n* = 37). In contrast, the multiple linear regression model predicting GTSC scores from physical, behavioral, and neurophysiological measures did not reach statistical significance (*F*(4, 26) = 2.713, *p* = .052, *R*^2^ = .294, adjusted *R*^2^ = .186), and none of the individual predictors was statistically significant. These findings suggest an association between inhibition-related ERP activity and response speed during soccer-specific decision making involving complex visual processing and lower-limb responses. Further studies using appropriate comparison groups and better-matched task conditions are needed to determine whether this relationship reflects soccer-specific expertise or more general differences in perceptual, cognitive, and motor demands.

## 1 Introduction

Soccer performance depends not only on physical fitness and technical skills but also on the ability to make rapid, appropriate decisions in dynamic game situations. Effective in-game decision-making requires players to select appropriate actions while simultaneously suppressing inappropriate or ineffective responses—a process referred to as inhibitory control. For example, the ability to withhold a pass that is no longer optimal because of a changing defensive configuration is a critical factor influencing overall performance quality. This process of active suppression, which halts a motor command before action execution, is a core component of executive function and is commonly assessed using Go/No-go tasks. Event-related potentials (ERPs), derived from time-locked averaging of electroencephalography (EEG) signals, provide high temporal resolution and serve as effective tools for electrophysiologically dissociating the cognitive processes underlying motor execution and inhibition. Compared with Go conditions, No-go trials are characterized by distinct ERP markers—specifically the N2 and P3 components—which typically peak over frontocentral regions within approximately 140–300 ms and 300–600 ms, respectively (1–4). The N2 component is generated from anterior midcingulate and inferior frontal sources, whereas the P3 component is associated with the precentral, middle frontal, and midcingulate regions (5). Previous reviews have comprehensively described the characteristics of the N2 and P3 components elicited during Go/No-go tasks. For example, auditory stimuli typically elicit substantially lower N2 and P3 amplitudes than visual stimuli (6, 7), indicating that these ERPs are markedly modulated by task sensory modality. Beyond sensory influences, inhibitory processing is also deeply intertwined with motor output. Previous studies have shown that stronger inhibitory neural activity is required to suppress a response during No-go stimulus, whereas stronger motor-related activation is required to execute an action during Go stimulus.

Furthermore, neuroelectric indices of inhibitory control are closely associated with behavioral outcomes, including muscle force production, reaction speed, and intraindividual response variability (8). Taken together, these findings indicate that inhibitory control is not limited to motor inhibition alone but is strongly integrated with motor execution processes.

Numerous studies have examined inhibitory control in athletes. For example, baseball players have shown enhanced No-go-N2 amplitudes compared with athletes in other sports. Furthermore, a positive relationship between No-go-N2 latency and reaction time (RT) has been reported (9). Similar increases in N2 amplitudes have also been observed in elite fencers (10). However, much of the existing research has relied on general, non-sport-specific tasks (e.g., somatosensory, auditory, and visual Go/No-go tasks). In contrast, evidence suggests that skilled baseball players conceptually integrate baseball-specific spatial information with response execution and inhibition (11). Therefore, to develop a precise understanding of motor execution and inhibitory processing in soccer players, reliance on standard stimulus paradigms alone is insufficient; soccer-specific tasks are essential. Furthermore, conventional cognitive tasks predominantly evaluate responses executed by the upper limbs (e.g., manual button presses). However, previous findings indicating sport- and effector-specific neural processing are not limited to upper-limb behavior but extend to broader motor systems. Recent evidence suggests that motor execution and inhibition are highly effector-dependent; specifically, lower-limb responses are associated with longer and more variable RTs, and although the upper and lower limbs share certain N2 and P3 characteristics, distinct ERP patterns emerge depending on limb type and left–right laterality (12). Given that soccer performance is fundamentally dependent on lower-limb actions, assessing foot-based responses is essential to ensure ecological validity. Consistent with this perspective, previous research using foot-response paradigms has shown that during soccer-specific Go/No-go tasks (e.g., pass-selection decisions), No-go-N2 amplitudes are uniquely associated with response speed and consistency (13). While these findings highlight sport- and effector-specific neural processing, soccer performance is not limited to reaction speed alone. Broader executive functions, including inhibitory control, are also closely associated with higher technical–tactical performance and physical fitness in athletes (14–16). For example, withholding a pass to avoid interception reflects tactical proficiency, and maintaining such rapid decision-making under high-intensity conditions requires adequate physical fitness. Despite these behavioral associations, empirical studies that integrate neurophysiological indices of inhibition (e.g., ERPs) with on-field physical and technical–tactical performance measures remain limited. Therefore, an integrated examination of these variables is necessary to clarify the role of inhibitory control in soccer performance.

The primary aim of this study was to examine the relationships among physical fitness, technical–tactical skills, and inhibitory ERP components in soccer players using auditory, visual, and soccer-specific Go/No-go tasks. Based on previous findings indicating that inhibitory ERP responses are influenced by stimulus modality and motor-response characteristics (9, 10), as well as evidence that inhibitory neural activity is associated with response speed (8), we hypothesized that the relationship between inhibitory ERP components—particularly the No-go-N2—and RT would differ across task conditions and would be more pronounced in the soccer-specific task than in the general auditory or visual tasks.

## 2 Materials and methods

### 2.1 Participants

Forty-one male amateur collegiate soccer players (20.7 ± 1.7 years) participated in this study. All participants were members of the official university team competing in the Japanese Kansai University Soccer 3rd League, representing a well-organized collegiate competitive level where players undergo regular, structured soccer training and official league matches. None reported any history of neurological or psychiatric disorders. Written informed consent was obtained from all participants prior to participation. All procedures were conducted in accordance with the Declaration of Helsinki for research involving human participants. Of the initial 41 participants, data from three participants could not be analysed because of technical problems during data acquisition, leaving 38 participants with valid behavioural data from the three Go/No-go tasks. For the ERP analyses, additional exclusions were made on a task-specific basis because eye-movement contamination resulted in fewer than 20 artifact-free trials available for ERP averaging. Specifically, one participant was excluded from the visual-task ERP analysis, three from the auditory-task ERP analysis, and one from the soccer-specific-task ERP analysis. Consequently, the ERP analyses included 37, 35, and 37 participants for the visual, auditory, and soccer-specific tasks, respectively. Physical-fitness assessments were completed by 32 participants, as several participants were unable to perform the tests because of minor injuries or related physical constraints. SSG/GTSC data were available for 38 participants. For the multiple linear regression analysis, listwise deletion was applied, and 31 participants with complete data for all variables entered into the model were included in the final analysis (Figure 1). This study was approved by the Ethical Committee of Osaka Metropolitan University, Osaka, Japan (Approval No. 22-65).

**Figure 1:**
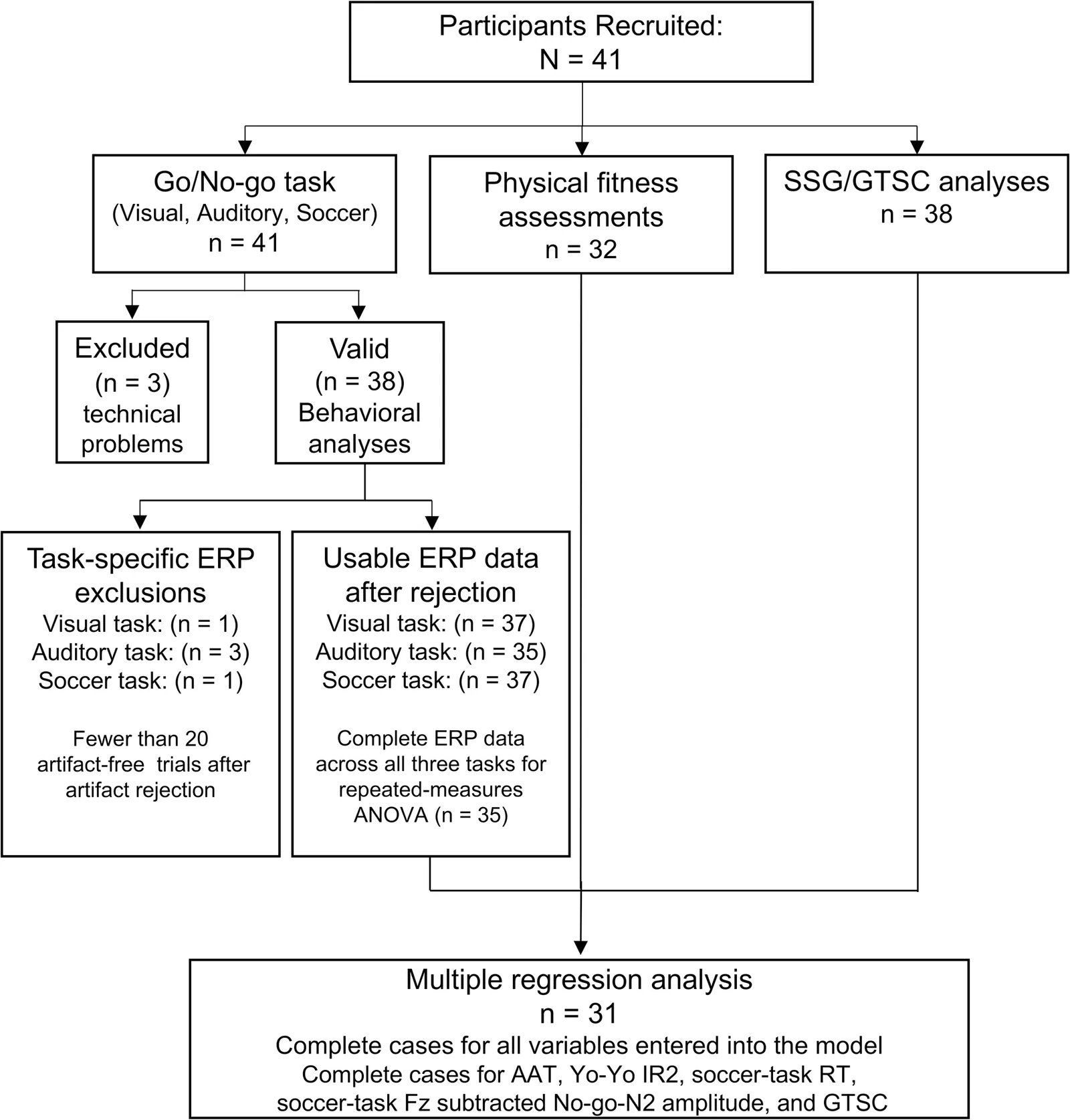
Participant flow and analytic sample sizes. Of 41 recruited players, 38 provided valid behavioral data for the Go/No-go tasks. ERP data were available for 37 participants in the visual and soccer-specific tasks and for 35 participants in the auditory task after artifact rejection. Physical-fitness and SSG/GTSC data were available for 32 and 38 participants, respectively, and 31 complete cases were included in the multiple regression analysis.

### 2.2 Physical fitness and technical–tactical skill tests

On-field performance was assessed using three physical fitness tests—the 30-m sprint, the Arrowhead Agility Test (AAT), and the Yo-Yo Intermittent Recovery Test Level 2 (Yo-Yo IR2)—as well as 4-vs-4 SSGs. Height, body mass, and body fat percentage were assessed before the physical-fitness tests. Height was measured using a stadiometer, whereas body mass and body fat percentage were measured using a multi-frequency body composition analyzer (MC-780A-N; TANITA Corporation, Tokyo, Japan). Data collection consisted of three assessment components conducted in the following chronological order: physical-fitness testing, EEG/Go/No-go testing, and 4-vs-4 SSG/GTSC assessment. Physical-fitness and EEG/Go/No-go assessments were conducted in March during the preseason period, with the physical-fitness assessment preceding the EEG/Go/No-go assessment. The SSG/GTSC assessment was conducted in June during the competitive season, approximately three months after the physical-fitness assessment. Participants were instructed to avoid strenuous exercise for at least 24 h before each assessment. During the competitive season, testing was conducted at least 48 h after a competitive match. Due to injuries and related constraints, the physical fitness tests were administered to 32 of the 41 participants. Following a standardized warm-up, participants received verbal instructions and demonstrations from the research team. The 30-m sprint and AAT were assessed using electronic timing gates (Brower Timing Systems, Draper, UT, USA, and Dashr, Dashr Motion Performance Systems, USA). Participants assumed a standing start position with their front foot aligned with the start line, initiating the sprint voluntarily rather than reacting to a signal. The AAT was conducted in accordance with previously described protocols (17), with a course of approximately 37 m that included diagonal and curved sprints. The best time from the two trials in each direction was recorded. Intermittent endurance was evaluated using the Yo-Yo IR2 (18), with total distance covered recorded as the performance outcome. These tests have been shown in multiple studies to be valid and reliable measures of soccer-specific physical fitness, particularly speed, change-of-direction ability, and intermittent recovery (17–20).

Technical–tactical performance was assessed using 4 vs. 4 SSGs, in which 38 of the 41 participants took part. The 4-vs-4 SSG format provides greater representativeness than isolated skill tests and is often more feasible than full-match assessment (21, 22). Each field measured 30 × 20 m and included two mini-goals (1 × 2 m). Participants played six 5-min matches with 2-min passive recovery periods between games. Game performance was quantified using total points derived from match outcomes and goals scored, as well as the Game Technical Scoring Chart (GTSC). The GTSC was used as a complementary method to subjectively quantify players’ technical–tactical performance. Ratings were based on coaches’ evaluation of individual performance across 10 soccer-specific criteria: assists, communication, control, cover/support, decision-making, first touch, marking, one-versus-one, passing, and shooting. Each criterion was scored on a 0–5 scale (1 = poor, 2 = below average, 3 = average, 4 = very good, and 5 = excellent), and a total score reflecting overall technical performance was calculated. GTSC ratings were conducted by four soccer coaches, each of whom held at least a Japan Football Association C license. All four coaches independently evaluated all players based on their performance during the SSGs and subsequent video review. The final GTSC score for each player was calculated as the mean of the four raters’ scores. Although the raters were familiar with the players, they were unaware of the participants’ physical-fitness, EEG, and Go/No-go results and were not involved in the collection of these data. Inter-rater reliability of the GTSC ratings was assessed using a two-way mixed-effects intraclass correlation coefficient with absolute agreement for the average of four raters. Previous research has shown that the GTSC is a valid and reliable tool for assessing the performance of academy soccer players (23, 24). In this study, total points and GTSC scores were analyzed separately as individual performance measures. GTSC scores were used in the correlation and multiple regression analyses.

### 2.3 Experimental procedure and tasks

Participants completed three Go/No-go tasks: a visual Go/No-go task (visual task), an auditory Go/No-go task (auditory task), and a soccer-specific pass-choice Go/No-go task (soccer task). In the visual Go/No-go task, visual stimuli were presented on a television monitor (LCD-AH221XDB-B, I-O DATA; 1920 × 1080 pixels) positioned approximately 1 m in front of the participants. The Go and No-go stimuli consisted of green and red circles, respectively, each presented for 100 ms at the center of the monitor against a black background. Go and No-go stimuli were presented in random order with equal probability. In the auditory Go/No-go task, stimuli were presented binaurally using headphones at 65 dB. The stimuli had a duration of 100 ms, with 10 ms rise and fall times. The Go and No-go stimuli were pure tones of 2,000 and 1,000 Hz, respectively, and were presented in random order with equal probability. In the soccer-specific pass-choice Go/No-go task, the Go and No-go stimuli consisted of images depicting five players (three attackers and two defenders) presented for 100 ms, as per a previously published protocol (13). The Go stimulus was an image depicting a pass directed toward the player positioned behind the two defenders. Participants were instructed to respond as quickly as possible by pressing a foot-switch button with their dominant foot only when a Go stimulus was presented. The No-go stimulus was an image depicting passes directed toward players positioned on either the left or right. The images depicted a first-person perspective of the ball holder during a 4-vs-2 possession-training scenario at the moment of pass-selection decision (Figure 2). The validity of the pass-selection images was confirmed by four licensed Japanese Football Association coaches, who independently reviewed all images. Twelve validated images were used in the task, including four Go images and eight No-go images. The interstimulus interval was fixed at 2,000 ms for all tasks. Each run comprised 80 stimulus trials: 40 Go and 40 No-go. Two runs were completed for each of the three conditions, resulting in a total of six runs. The order of the task conditions was randomized within participants and counterbalanced across participants. Before data collection, participants completed a practice session consisting of 10 stimulus trials to familiarize themselves with the tasks.

**Figure 2:**
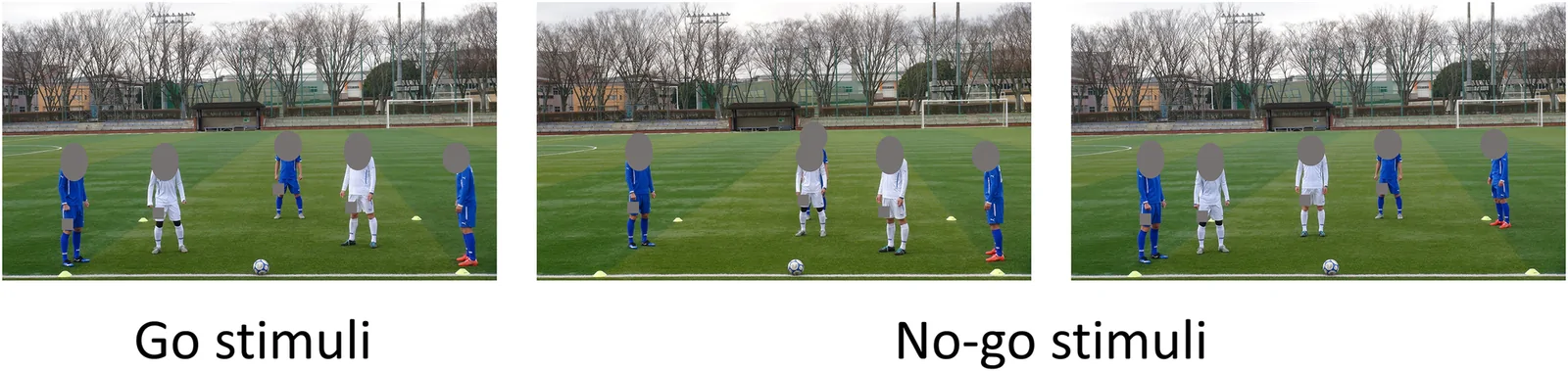
Representative images used in the soccer-specific pass-choice Go/No-go task. The stimuli consisted of first-person perspective images depicting a 4-vs-2 possession-training scenario with five players (three attackers and two defenders) at the moment of pass-selection decision. Go stimuli depicted a passing option toward the player positioned behind the two defenders and required participants to press a foot-switch button. No-go stimuli depicted passing options toward a player positioned on either the left or right side and required participants to inhibit their response. A total of 12 approved images were used in the task (4 Go and 8 No-go stimuli).

### 2.4 EEG recording

EEG activity was recorded using Ag/AgCl disk electrodes positioned at the Fz, Cz, and Pz sites according to the international 10–20 system. Signals from each scalp electrode were referenced to the linked earlobes and subsequently recalculated using an average reference. Electrooculographic activity was bipolarly recorded to identify and reject trials contaminated by eye movements or blinks exceeding 100 *μ*V. For this purpose, a pair of electrodes was placed 2 cm lateral to the lateral canthus of the left eye and 2 cm above the upper margin of the left orbit. Furthermore, all raw EEG data were visually inspected offline, and trials contaminated by eye movements or blinks exceeding ±100 μV were excluded from ERP averaging. A minimum of 20 artifact-free trials was required for ERP averaging in each task condition. Participants for whom fewer than 20 artifact free trials remained after artifact rejection were excluded from the ERP analysis. Electrode impedance was maintained below 5 k*Ω* throughout data acquisition. All EEG signals were recorded using a signal processor (MaP1200b; NIHON SANTEKU Co., Ltd., Osaka, Japan). Epochs used for ERP analysis extended to 700 ms following stimulus onset, with the 200-ms prestimulus interval used as the baseline. The bandpass filter was set to 0.33–30 Hz, and data were sampled at 1000 Hz. The mean numbers of accepted trials for the Go and No-go trials were 62.5 ± 15.0 and 51.7 ± 19.6, respectively, in the visual task; 66.3 ± 12.1 and 56.4 ± 17.2, respectively, in the auditory task; and 60.1 ± 13.4 and 50.1 ± 15.4, respectively, in the soccer task. No systematic differences in trial loss were observed between the Go and No-go trials or across task conditions.

To isolate the No-go-specific neural activity, difference waveforms were calculated by subtracting the ERP elicited during Go trials from that elicited during No-go trials, thereby minimizing the overlapping activity shared between Go and No-go ERPs (9, 25, 26). The resulting difference waveform exhibited two prominent peaks. For the ERP analyses, peak amplitudes and latencies of the N2 and P3 components in the difference waveforms were quantified within time windows of 170–320 ms and 260–500 ms, respectively. These temporal windows were applied uniformly across all conditions (Visual, Auditory, and Soccer), following previous literature (27). Amplitudes were quantified from baseline to peak, and these time windows were applied consistently across all conditions and participants. Peak amplitudes and latencies of the N2 and P3 components were determined in Microsoft Excel by identifying the most negative peak for the N2 component and the most positive peak for the P3 component within the predefined latency windows. All identified peaks were then verified through visual inspection.

### 2.5 Behavioral data

Behavioral data included the RT, the standard deviation (SD) of RT as an index of reaction-time variability, omission errors (missed or delayed responses to Go stimuli), and commission errors (incorrect responses to No-go stimuli). Following previous Go/No-go ERP studies (28), the omission error rate included both failures to respond during Go trials and responses occurring more than 800 ms after stimulus presentation. The overall error rate was calculated as the average of the commission and omission error rates, as the primary focus of the analysis was overall task accuracy rather than distinctions between error types.

### 2.6 Statistical analysis

Behavioral data, including mean RT, the SD of RT, and omission and commission error rates, were analyzed using a repeated-measures ANOVA with task condition (visual, auditory, and soccer) as a within-subject factor. The amplitudes and latencies of the subtracted No-go-N2 and subtracted No-go-P3 components were analyzed separately using a two-way repeated-measures ANOVA, with task condition and electrode site (Fz, Cz, and Pz) as within-subject factors. When Mauchly's test indicated a violation of the sphericity assumption, Greenhouse–Geisser corrections were applied to adjust the degrees of freedom using the epsilon (*ε*) correction factor. Bonferroni-adjusted *post hoc* comparisons were performed to assess differences among task conditions.

Bivariate correlations were subsequently analyzed among physical fitness, technical–tactical performance scores, behavioral data, and ERP components (i.e., subtracted No-go-N2 and No-go-P3 amplitudes). Data normality was assessed using the Kolmogorov–Smirnov test. Pearson's correlation coefficients were calculated for normally distributed variables, whereas Spearman's correlations were used for non-normally distributed data. Benjamini–Hochberg false discovery rate (FDR) correction was applied separately within each task condition, with an FDR threshold of *q* < .05. Each task-specific family comprised 36 correlations: six ERP measures (subtracted No-go-N2 and No-go-P3 amplitudes at Fz, Cz, and Pz) were correlated with six performance or behavioral measures (30-m sprint, AAT, Yo-Yo IR2, GTSC score, task-specific RT, and task-specific RT variability). Thus, 36 correlations were included in each of the visual, auditory, and soccer-specific task families. The task conditions were treated as separate families because the analyses focused on task-dependent relationships between ERP activity and behavioral/performance measures, and correlations between ERP measures and behavioral measures from different task modalities were not examined. As a sensitivity analysis, the Benjamini–Hochberg procedure was additionally applied across all 108 correlations from the three task conditions.

To explore independent associations of physical, behavioral, and neurophysiological measures with GTSC scores, a multiple linear regression analysis was performed using the enter method. Given the limited complete-case sample size (*n* = 31), the regression analysis was considered exploratory, and the number of predictors was restricted to four to avoid overparameterization. Predictor selection was exploratory rather than prespecified *a priori*. Among the physical-fitness measures, the AAT and Yo-Yo IR2 were selected to represent complementary aspects of soccer-related fitness, namely change-of-direction ability and intermittent endurance, respectively. The 30-m sprint was excluded to reduce conceptual redundancy among the physical-fitness predictors because the AAT also includes a substantial sprinting component. Soccer-task RT and Fz subtracted No-go-N2 amplitude were included as behavioral and neurophysiological variables relevant to the study aim of examining the relationship between inhibitory processing and soccer-related performance. Accordingly, the AAT, Yo-Yo IR2, soccer-task RT, and Fz subtracted No-go-N2 amplitude were entered simultaneously into the model. Multicollinearity was assessed using the variance inflation factor (VIF), with values > 10 considered indicative of problematic multicollinearity. Statistical significance was set at *p* < 0.05.

## 3 Results

### 3.1 Behavioral data

Table 1 presents anthropometric characteristics, physical fitness tests, and technical–tactical performance. Inter-rater reliability for the mean GTSC score across the four raters was excellent (ICC = 0.916, 95% CI (0.858, 0.953), *p* < 0.001). Table 2 presents behavioral data, including RT, the SD of RT, and omission and commission error rates. For RT, Mauchly's test indicated a violation of the assumption of sphericity (*W* = 0.697, *p* = 0.002); therefore, degrees of freedom were corrected using Greenhouse Geisser estimates (*ε* = 0.768). The repeated-measures ANOVA revealed a significant main effect of task (*F*(1.5, 56.8) = 102.414, *p* < 0.001, *η^2^* = 0.735). *Post hoc* Bonferroni comparisons indicated that the RT was significantly longer in the soccer task than in both the visual and auditory tasks (*p* < 0.01 for both comparisons). Regarding the SD of RT, the main effect of task was significant (*F*(2, 74) = 31.665, *p* < 0.001, *η^2^* = 0.461). *Post hoc* Bonferroni tests showed that the SD of RT was significantly longer in the soccer task than in the visual and auditory tasks (*p* < 0.01 and *p* < 0.05, respectively), and higher in the auditory task than in the visual task (*p* < 0.01). For omission errors, there was a significant main effect of task (*F*(2, 74) = 8.638, *p* < 0.001, *η^2^* = 0.189). *Post hoc* Bonferroni comparisons indicated that omission errors were significantly higher in the soccer task than in both the visual and auditory tasks (*p* < 0.05 for both comparisons). For commission errors, Mauchly's test indicated a violation of the sphericity assumption (*W* = 0.219, *p* < 0.001); therefore, the Greenhouse–Geisser correction was applied (*ε* = 0.562). The repeated-measures ANOVA revealed a significant main effect of task (*F*(1.1, 41.5) = 39.529, *p* < 0.001, *η^2^* = 0.517). *Post hoc* Bonferroni comparisons showed that commission errors were significantly higher in the soccer task than in both the visual and auditory tasks (*p* < 0.01 for both comparisons).

**Table 1:** Anthropometric characteristics, physical fitness tests, and technical–tactical performance (mean SD).

Age (years)	20.7 (1.7)
Height (cm)	171.6 (4.8)
Weight (kg)	63.9 (5.2)
Body fat (%)	10.6 (2.8)
Physical fitness test	
30-m sprint (s)	4.3 (0.2)
AAT (s)	8.3 (0.2)
Yo-Yo IR2 (m)	640 (133.7)
Technical–tactical performance data	
GTSC score (points)	25.9 (6.3)
Total point (points)	11.7 (5.6)

**Table 2:** Behavioral data (mean SD).

	Visual	Auditory	Soccer
RT (ms)	328 (40)**	323 (65)**	400 (49)
SD of RT (ms)	46 (16)**,***	58 (17)*	69 (18)
Omission errors (%)	0.5 (0.8)*	0.5 (1.1)*	1.4 (1.6)
Commission errors (%)	0.4 (0.5)**	0.6 (0.7)**	3.1 (2.7)

* vs. Soccer *p* < 0.05.

** vs. Soccer *p* < 0.01.

*** vs. Auditory *p* < 0.05.

Figure 3A shows the grand-averaged ERP waveforms for the Go and No-go trials across all task conditions, whereas Figure 3B shows the corresponding grand-averaged difference waveforms. In the visual and soccer tasks, clearly subtracted No-go-N2 and No-go-P3 components were observed. In contrast, the auditory task exhibited a relatively smaller subtracted No-go-N2 amplitude. The mean amplitudes and latencies of the subtracted No-go-N2 and No-go-P3 components are presented in Table 3.

**Figure 3:**
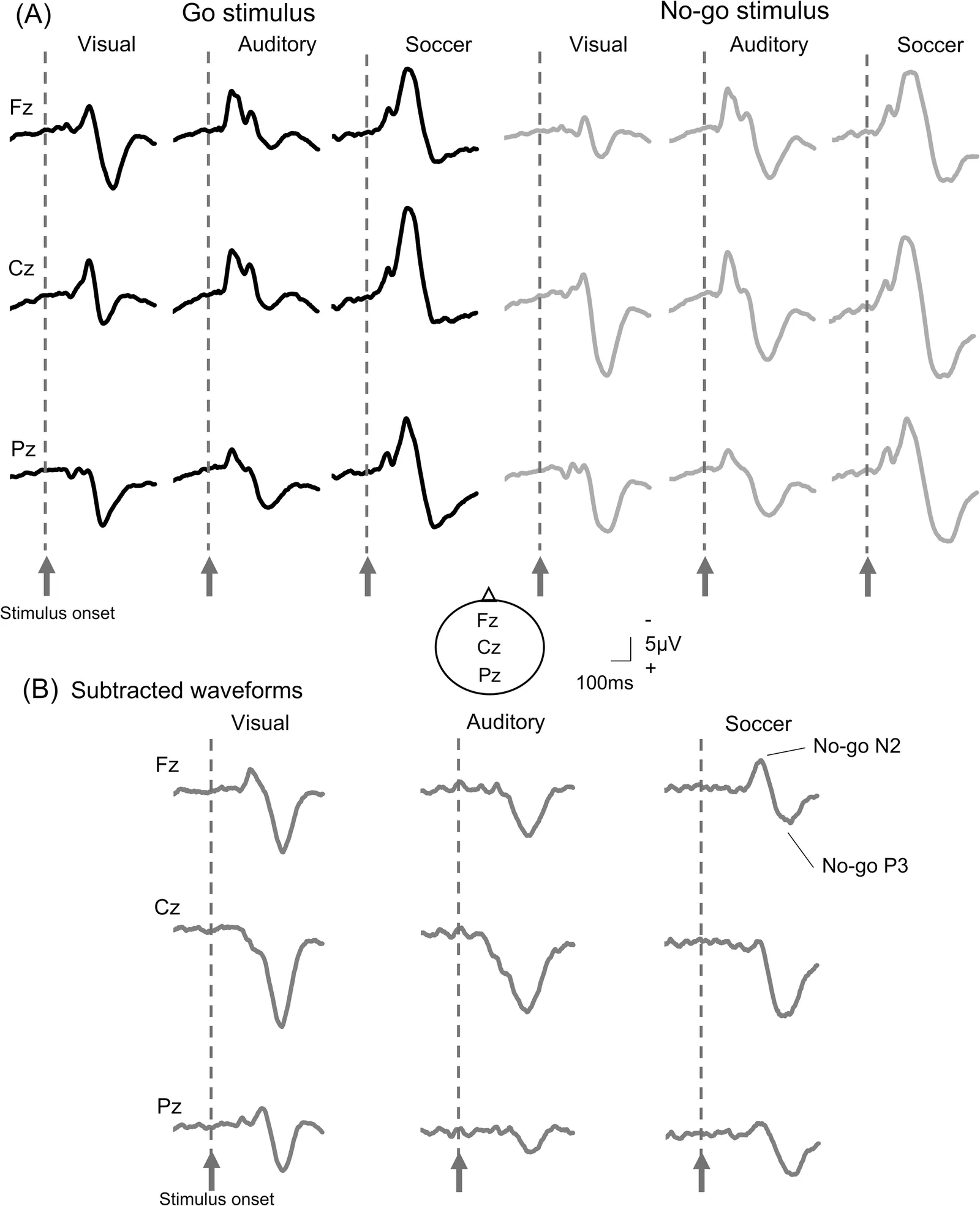
**(A)** Grand-averaged ERP waveforms for the visual, auditory, and soccer-specific Go/No-go tasks, elicited by go (black line) and no-go (gray line) stimuli. **(B)** Grand-averaged subtracted waveforms (No-go minus Go).

**Table 3:** Mean (SD) of subtracted No-go-N2 and No-go-P3 amplitudes and latencies at Fz, Cz, and Pz electrodes during visual, auditory, and soccer-specific Go/No-go tasks.

		Visual	Auditory	Soccer
Subtracted No-go-N2 amplitude (μV)	Fz	−5.0 (2.7)^a^^,^^c^	−2.9 (3.1)^c^	−5.6 (3.1)^a^^,^^c^
	Cz	−2.7 (2.8)^a^	−0.51 (3.2)	−3.6 (2.7)^a^
	Pz	−5.8 (3.5)^a^^,^^c^	−4.5 (3.8)^c^	−4.3 (2.7)
Subtracted No-go-N2 latency (ms)	Fz	225 (35)^b^	218 (41)^b^	285 (50)
	Cz	210 (54)^b^	205 (55)^b^	253 (63)^d^
	Pz	236 (52)^c^	234 (57)^c^	266 (62)
Subtracted No-go-P3 amplitude (μV)	Fz	9.4 (4.8)^b^^,^^c^	8.2 (4.2)^b^^,^^c^	6.0 (4.0)^c^
	Cz	15.0 (7.1)^a^	13.1 (6.3)	12.6 (6.0)
	Pz	7.1 (4.9)^a^^,^^c^	5.1 (5.3)^c^^,^^d^	7.0 (4.6)^a^^,^^c^
Subtracted No-go-P3 latency (ms)	Fz	381 (31)	372 (47)	406 (51)
	Cz	369 (37)	359 (47)	413 (36)
	Pz	392 (32)	368 (55)	411 (55)

All *post-hoc* comparisons were Bonferroni-adjusted (*p* < .05).

a Significant difference vs. Auditory.

b significant difference vs. Soccer.

c significant difference vs. Cz.

d significant difference vs. Fz.

### 3.2 Amplitude and latency of the subtracted No-go-N2 and subtracted No-go-P3

Table 3 presents ERP data for the amplitudes and latencies of the subtracted No-go-N2 and subtracted No-go-P3 components. For the subtracted No-go-N2 amplitude, the two-way repeated-measures ANOVA revealed significant main effects of task (*F*(2, 68) = 7.657, *p* = 0.001, *η^2^* = 0.184) and, electrode (*F*(2, 68) = 34.880, *p* < 0.001, *η^2^*= 0.506). Furthermore, a significant task–electrode interaction was observed (*F*(3.16, 107.67) = 8.286, *p* < 0.001, *η^2^*= 0.196). Degrees of freedom were corrected using Greenhouse–Geisser estimates due to a violation of sphericity (*W* = 0.578, *p* = 0.038, *ε* = 0.792). Bonferroni-corrected *post hoc* tests indicated that amplitudes at Fz and Cz were significantly larger in the visual and soccer tasks than in the auditory task (*p* < 0.05). Additionally, the amplitude at Pz was significantly larger in the visual task than in the auditory task (*p* < 0.05). In the visual and auditory tasks, amplitudes were significantly larger at Fz and Pz than at Cz (*p* < 0.01), whereas in the soccer task, the amplitude at Fz was significantly larger than at Cz (*p* < 0.01).

For the latency of the subtracted No-go-N2 component, the ANOVA revealed significant main effects of task (*F*(1.66, 56.65) = 17.399, *p* < 0.001, *η^2^*= 0.339) and electrode (*F*(2, 68) = 8.092, *p* < 0.001, *η^2^*= 0.192). Given that Mauchly's test indicated a violation of the sphericity assumption (*W* = 0.800, *p* = 0.025), Greenhouse–Geisser corrections were applied (*ε* = 0.833). A significant task–electrode interaction was also observed (*F*(3.09, 105.35) = 2.798, *p* = 0.028, *η^2^*= 0.076). Greenhouse–Geisser correction was similarly applied to the interaction effect because of a violation of sphericity (*W* = 0.520, *p* = 0.012, *ε* = 0.775). *Post hoc* analyses of the interaction revealed that latencies at Fz and Cz were significantly shorter in the visual and auditory tasks than in the soccer task (*p* < 0.01). Furthermore, latencies in the visual and auditory tasks were significantly shorter at Cz than at Pz (*p* < 0.01), whereas in the soccer task, latency at Cz was significantly shorter than at Fz (*p* < 0.05).

For the amplitude of the subtracted No-go-P3, the results indicated significant main effects of task (*F*(2, 68) = 5.019, *p* = 0.009, *η^2^*= 0.129) and electrode site (*F* (2, 68) = 77.268, *p* < 0.001, *η^2^*= 0.694), as well as a significant task–electrode interaction (*F*(4, 136) = 11.779, *p* < 0.001, *η^2^*= 0.257). *Post hoc* comparisons indicated that the amplitude at Fz was significantly larger in the visual and auditory tasks than in the soccer task (*p* < 0.05). At Cz amplitudes were significantly larger in the visual task than in the auditory task (*p* < 0.05). Conversely, at Pz amplitudes were significantly larger in both the visual and soccer tasks than in the auditory task (*p* < 0.05). Across all tasks, amplitudes were significantly larger at Cz than at Fz and Pz (*p* < 0.01). Finally, in the auditory task, the amplitude at Fz was significantly larger than at Pz (*p* < 0.01).

Regarding the latency of the subtracted No-go-P3 component, the ANOVA revealed a significant main effect of task (*F*(2, 68) = 15.524, *p* < 0.001, *η^2^*= 0.313). *Post hoc* tests showed that latency was significantly shorter in the auditory task than in the soccer task (*p* < 0.001). There was no significant main effect of electrode site (*F* (2, 68) = 1.661, *p* = 0.198, *η^2^*= 0.047), and the task–electrode interaction was also not significant (*F*(2.86, 97.37) = 1.409, *p* = 0.246, *η^2^*= 0.040). Degrees of freedom for the interaction term were corrected using Greenhouse–Geisser estimates due to a violation of sphericity (*W* = 0.493, *p* = 0.006, *ε* = 0.716).

### 3.3 Relationship between physical fitness, technical–tactical performance, and ERP components

Tables 4, 5 present the correlation coefficients (*r*) describing the relationships among physical fitness measures, technical–tactical performance scores, behavioral data, and ERP components across tasks. During the soccer task, a significant correlation was observed between RT and the peak amplitude of the subtracted No-go-N2 component at Fz (*r* = 0.552, *p* < 0.001, *n* = 37), indicating that shorter RTs were associated with more negative peak amplitudes of the subtracted No-go-N2 component (Figure 4). This association remained significant after the task-specific Benjamini–Hochberg FDR correction (*q* = 0.014). In the sensitivity analysis applying the FDR correction across all 108 correlations, the association also remained significant (*q* = 0.043). No other correlations remained significant after FDR correction.

**Table 4:** Correlation coefficients between physical fitness, technical–tactical performance, behavioral data, and subtracted No-go-N2 amplitudes across visual, auditory, and soccer-specific Go/No-go tasks.

		30-m (s)	AAT (s)	Yo-Yo (m)	GTSC (points)	Visual RT (ms)	Auditory RT (ms)	Soccer RT (ms)	Visual SD (ms)	Auditory SD (ms)	Soccer SD (ms)
Visual tasks Subtracted No-go-N2 amplitude (μV) ERP available: *n* = 37 Paired *n*: fitness = 32; GTSC/behavioral =37	Fz	−0.017	0.025	0.091^a^	0.195	0.288	-	-	0.185^a^	-	-
	Cz	−0.096	−0.037	0.205^a^	0.162	−0.029	-	-	0.080^a^	-	-
	Pz	−0.04	−0.091	−0.074^a^	0.18	0.227	-	-	0.143^a^	-	-
Auditory tasks Subtracted No-go-N2 amplitude (μV) ERP available: *n* = 35 Paired *n*: fitness = 32; GTSC/behavioral =35	Fz	−0.004	−0.097	−0.061^a^	−0.02	-	0.283^a^	-	-	0.212	-
	Cz	−0.047	−0.295	0.009^a^	0.113	-	0.014^a^	-	-	0.018	-
	Pz	−0.193^a^	−0.337^a^	0.039^a^	0.243^a^	-	0.335^a^	-	-	0.251^a^	-
Soccer tasks Subtracted No-go-N2 amplitude (μV) ERP available: *n* = 37 Paired *n*: fitness = 32; GTSC/behavioral =37	Fz	0.244	0.23	−0.382^a^	−0.227	-	-	0.552*	-	-	0.198
	Cz	0.065	0.057	−0.161^a^	−0.385	-	-	0.461	-	-	0.042
	Pz	−0.118	−0.045	0.104^a^	−0.196	-	-	0.380	-	-	0.142

Dashes (-) indicate that correlations were not calculated between different modalities.

Sample sizes varied according to data availability and are reported for each task-specific block of correlations.

a Spearman's rank correlation coefficient; all other values are Pearson's correlation coefficients.

* *p* < 0.05.

**Table 5:** Correlation coefficients between physical fitness, technical–tactical performance, behavioral data, and subtracted No-go-P3 amplitudes across visual, auditory, and soccer-specific Go/No-go tasks.

		30-m (s)	AAT (s)	Yo-Yo (m)	GTSC (points)	Visual RT (ms)	Auditory RT (ms)	Soccer RT (ms)	Visual SD (ms)	Auditory SD (ms)	Soccer SD (ms)
Visual tasks Subtracted No-go-P3 amplitude (μV) ERP available: *n* = 37 Paired *n*: fitness = 32; GTSC/behavioral =37	Fz	−0.141	−0.118	0.080^a^	−0.015	−0.229	-	-	−0.235^a^	-	-
	Cz	−0.191	−0.226	0.336^a^	0.18	−0.084	-	-	−0.155^a^	-	-
	Pz	−0.013	−0.12	0.265^a^	0.136	−0.133	-	-	−0.055^a^	-	-
Auditory tasks Subtracted No-go-P3 amplitude (μV) ERP available: *n* = 35 Paired *n*: fitness = 32; GTSC/behavioral =35	Fz	−0.183^a^	−0.235^a^	0.171^a^	0.05^a^	-	−0.123^a^	-	-	−0.016^a^	-
	Cz	−0.255	−0.387	0.316^a^	0.369	-	−0.027^a^	-	-	0.025	-
	Pz	−0.156	−0.203	0.181^a^	0.239	-	0.030^a^	-	-	−0.004	-
Soccer tasks Subtracted No-go-P3 amplitude (μV) ERP available: *n* = 37 Paired *n*: fitness = 32; GTSC/behavioral =37	Fz	−0.198	−0.27	0.030^a^	0.189	-	-	−0.205	-	-	−0.044
	Cz	−0.224	−0.291	0.243^a^	0.448	-	-	−0.101	-	-	0
	Pz	−0.108	−0.182	0.229^a^	0.393	-	-	−0.096	-	-	0.043

Sample sizes varied according to data availability and are reported for each task-specific block of correlations.

a Spearman's rank correlation coefficient; all other values are Pearson's correlation coefficients.

**Figure 4:**
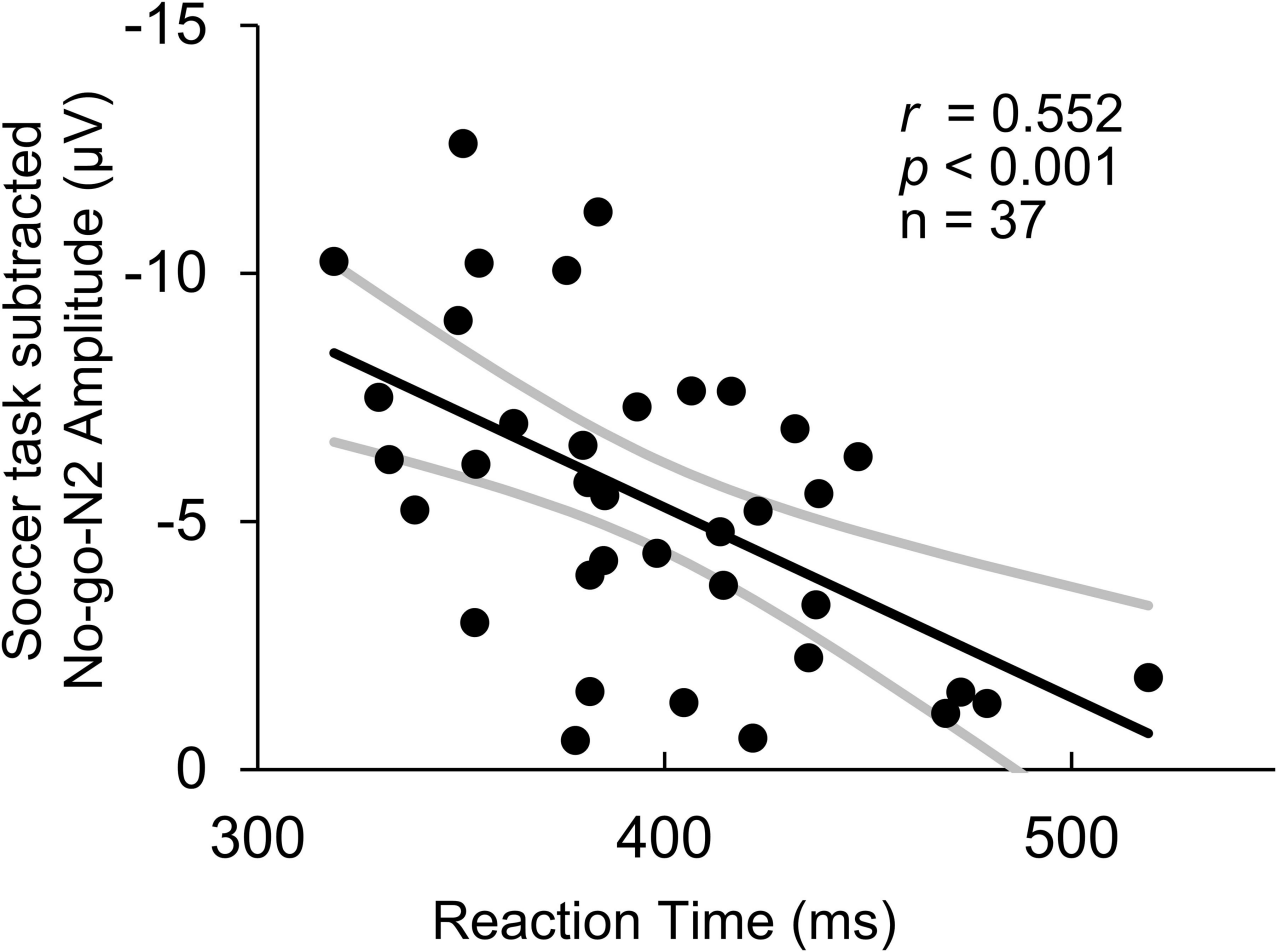
Relationship between reaction time (RT) and the peak amplitude of the subtracted No-go-N2 at Fz during the soccer-specific Go/No-go task (*n* = 37). The solid black line represents the fitted regression line, and the gray lines indicate the 95% confidence interval around the regression line.

### 3.4 Multiple linear regression analysis

Table 6 presents the results of the multiple regression analysis. The overall model was not statistically significant (*F*(4, 26) = 2.713, *p* = 0.052, with *R^2^* = 0.294, adjusted *R^2^* = 0.186). None of the individual predictors was statistically significant (Yo-Yo IR2: *β* = 0.349, *p* = 0.090; soccer-task: *β* = 0.218, *p* = 0.275; AAT: *β* = −0.237, *p* = 0.231; subtracted No-go-N2 at Fz: *β* = −0.148, *p* = 0.470).　The reduction from *R^2^* to adjusted *R^2^* indicates that the explanatory value of the model was substantially attenuated after accounting for the number of predictors and sample size.

**Table 6:** Multiple linear regression analysis predicting technical–tactical performance (GTSC) from physical-fitness, behavioral, and neurophysiological measures.

	*B*	SE	*β*	t	*p*	Tolerance	VIF
Arrowhead agility test (s)	−8.428	6.871	−0.237	−1.227	0.231	0.728	1.374
Yo-Yo IR2 (m)	0.015	0.009	0.349	1.761	0.090	0.691	1.446
Soccer RT (ms)	0.029	0.026	0.218	1.115	0.275	0.712	1.404
Soccer tasks Fz subtracted No-go-N2 amplitude (μV)	−0.288	0.393	−0.148	−0.733	0.470	0.669	1.494

*n* = 31. *R^2^* = 0.294; adjusted *R^2^* = 0.186; *F*(4, 26) = 2.713, *p* = 0.052.

B, unstandardized regression coefficient; SE, standard error; β, standardized regression coefficient; VIF, variance inflation factor.

## 4 Discussion

Although previous studies have examined the relationship between executive functions and athletic performance (14–16), to the best of our knowledge, this is the first study to examine the integrated relationship among physical fitness, technical–tactical skills, and neurophysiological indices of inhibitory control in soccer players using a soccer-specific, foot-based Go/No-go task. In line with our hypothesis, this study revealed a marked correlation between RT and the subtracted No-go-N2 amplitude during the soccer-specific task. Previous studies have shown that the N2 amplitude reflects early stimulus processing at a premotor level, involving conflict monitoring and the suppression of prepotent responses. Within the Go/No-go paradigm, the No-go-N2 is widely regarded as a neurophysiological index of early, modality-dependent inhibitory control (2, 5, 6).

Previous studies have shown that baseball players and elite fencers exhibit larger No-go-N2 amplitudes during Go/No-go tasks and show greater modulation of sensorimotor inhibitory responses to the No-go stimulus (9, 10). Importantly, these N2 modulations do not merely reflect sensory processing. Rather, they represent early inhibitory mechanisms that support rapid decision-making in complex situations, specifically the active cancellation of motor responses.

To accurately capture these sport-specific inhibitory mechanisms, a highly ecologically valid soccer task was employed in this study. The ERP results reflected the unique cognitive demands of this paradigm. Specifically, the subtracted No-go-N2 amplitude in the soccer task was markedly larger at the frontocentral electrodes (Fz and Cz) than in the auditory task, with a pronounced frontal maximum (Fz > Cz). This frontocentral distribution is consistent with previous Go/No-go studies reporting larger No-go N2 activity over frontocentral scalp regions and may reflect greater engagement of inhibitory and conflict-related processing during the soccer-specific task. Conversely, the subtracted No-go-N2 latency was markedly longer in the soccer task than in the visual and auditory tasks. This delay likely reflects the additional processing time required for complex visuospatial discrimination, such as evaluating the positioning of defenders and teammates, before initiating an early inhibitory response. Furthermore, the subtracted No-go-P3 component, which reflects later-stage inhibitory processing and stimulus evaluation (1, 5), was maximal at the Cz electrode across all tasks. Although the soccer task elicited a smaller P3 amplitude at Fz than the visual and auditory tasks, its latency was markedly longer than that of the auditory task, further indicating the high cognitive demands associated with evaluating soccer-specific visual scenes. The findings of this study extend this area of research by indicating that the relationship between inhibitory neural activity and response speed—previously reported primarily using general visual and auditory stimuli with upper-limb (manual) responses—is also evident when soccer-specific visual information is combined with lower-limb (foot-based) motor responses. A previous functional magnetic resonance imaging study using a somatosensory Go/No-go task reported that neural activity was stronger during No-go trials than during Go trials in the dorsolateral prefrontal cortex, ventrolateral prefrontal cortex, anterior cingulate cortex, inferior parietal lobule, and caudate (29). Although the present EEG study cannot directly infer the involvement of specific cortical or subcortical structures, this finding provides a useful conceptual framework for interpretation. Importantly, a significant association between RT and the subtracted No-go-N2 amplitude was observed exclusively during the soccer-specific task, with shorter RTs associated with more negative No-go-N2 amplitudes at Fz. This finding suggests that faster lower-limb responses to soccer-specific visual cues may be associated with greater engagement of early inhibitory or conflict-related processing. However, because the soccer-specific task required more complex visuospatial discrimination and decision making and was behaviorally more demanding than the general visual and auditory tasks, this association cannot be attributed specifically to soccer expertise. Rather, greater task difficulty, stimulus complexity, and perceptual–motor demands may also have contributed to the observed relationship. Thus, the task-specific RT–N2 association may reflect differences in task demands across modalities, which may influence the relationship between ERP activity and behavioral performance in soccer players.

Interestingly, although the No-go-N2 amplitude was associated with foot-based RT during the soccer-specific task, these cognitive–motor indices did not independently predict technical–tactical performance (GTSC). This finding suggests that the relationship between inhibitory-related ERP activity and rapid motor responses may not directly translate into broader tactical performance, which is likely determined by multiple interacting physical, technical, and cognitive factors. Given that the regression model did not reach statistical significance, the observed contribution of physical-fitness measures should be interpreted as exploratory rather than as evidence of an independent or foundational role in technical–tactical performance.

### 4.1 Practical implications

The findings of this study also offer valuable insights into soccer training and performance assessments. First, our results suggest that utilizing soccer-related visual cues and foot-based actions may capture specific cognitive–motor processes relevant to the sport. Therefore, practitioners may benefit from moving beyond generalized computer-based cognitive tests. Cognitive–motor training should more directly integrate soccer-specific perceptual decision-making with lower-limb execution, such as reactive passing drills or SSGs. Second, the present regression analysis did not identify any physical or cognitive measure as an independent predictor of technical–tactical performance. Therefore, specific training recommendations regarding intermittent endurance cannot be derived from the present regression findings. Nevertheless, the observed coefficient pattern may provide a hypothesis for future studies examining how physical, cognitive, and neurophysiological factors jointly contribute to on-field performance.

### 4.2 Limitations and future research

This study has several limitations. First, the relatively small sample size may limit the generalizability of the findings and reduce statistical power, particularly for the multiple regression analysis. In addition, because the sample consisted solely of male athletes, the findings may not generalize to female athletes. Second, because the study included only soccer players and no non-player, novice, or other-sport comparison group, the task-specific RT–No-go-N2 association cannot be attributed specifically to soccer expertise or training-induced neural adaptation. Third, EEG recordings were restricted to midline electrodes (Fz, Cz, and Pz), which precludes precise neural source localization. Fourth, previous validation studies of the GTSC have primarily involved youth or academy soccer players, and its validity in adult collegiate players has not been fully established, although excellent inter-rater reliability was observed in the present sample. Fifth, the physical-fitness and EEG/Go/No-go assessments were conducted during the preseason, whereas the SSG/GTSC assessment was conducted approximately three months later during the competitive season. This interval may have introduced temporal variability in physical fitness and technical–tactical performance. Sixth, foot-based RT and inhibitory control were evaluated using two-dimensional images in a controlled laboratory setting, which may not fully capture the dynamic perceptual, physical, and contextual demands of actual soccer play. Future studies with larger and more diverse samples, appropriate comparison groups, and more ecologically valid task conditions are needed to examine how expertise, sex, and sport-specific demands influence the relationships among neurophysiological, physical, and technical–tactical measures.

## 5 Conclusion

In conclusion, the findings of this study indicate that a significant association between No-go-N2 amplitude and lower-limb RT was observed during the soccer-specific task. However, the multiple regression model predicting technical–tactical performance did not reach statistical significance, and none of the individual predictors independently predicted GTSC. These findings suggest that the relationship between inhibitory-related neural activity and response speed may depend on task demands, whereas the contributions of physical and cognitive factors to technical–tactical performance remain to be clarified.

## Data Availability

The original contributions presented in the study are included in the article/Supplementary Material, further inquiries can be directed to the corresponding author.
